# The state of the art in peer review

**DOI:** 10.1093/femsle/fny204

**Published:** 2018-08-23

**Authors:** Jonathan P Tennant

**Affiliations:** 27 Paget Street, Aylestone, Leicester, UK, LE2 8SP

**Keywords:** peer review, scholarly communication, publishing, web 2.0, social networks

## Abstract

Scholarly communication is in a perpetual state of disruption. Within this, peer review of research articles remains an essential part of the formal publication process, distinguishing it from virtually all other modes of communication. In the last several years, there has been an explosive wave of innovation in peer review research, platforms, discussions, tools and services. This is largely coupled with the ongoing and parallel evolution of scholarly communication as it adapts to rapidly changing environments, within what is widely considered as the ‘open research’ or ‘open science’ movement. Here, we summarise the current ebb and flow around changes to peer review and consider its role in a modern digital research and communications infrastructure and suggest why uptake of new models of peer review appears to have been so low compared to what is often viewed as the ‘traditional’ method of peer review. Finally, we offer some insight into the potential futures of scholarly peer review and consider what impacts this might have on the broader scholarly research ecosystem. In particular, we focus on the key traits of certification and reputation, moderation and quality control and engagement incentives, and discuss how these interact with socio-technical aspects of peer review and academic culture.

## Teaser

The state of the art in scholarly peer review.

## INTRODUCTION

Peer review is one of the strongest social constructs within the self-regulated world of academia and scholarly communication. Researcher attitudes towards peer review are often in a state of reverence hailing it as a ‘golden standard’ (Mayden 2012; D'Andrea, James and O'Dwyer 2017), and even sometimes the distinction between a binary state of ‘verified’ and ‘unverified’ for research papers published in scholarly journals. Having a piece of research, including articles, books, conference proceedings and even grant applications, attain the status of ‘peer reviewed’ is considered to be a defining moment in the career of any scholar, and it has an incredible amount of capital attributed to it for scholarly reputation. Peer review is purported to serve many functions, including quality control as a screening mechanism, legitimisation of scientific research and the self-regulation of scientific communities. As such, in modern academia peer review remains critical in defining professional advancement and the hierarchical structure of research institutes (Fyfe et al. 2017; Moore et al. 2017), and is generally held in high regard across research communities (Goodman 1994; Bedeian 2003; Ware, 2011, 2015; Pierson 2018; Jutta andFredrik 2016).

With so much standing attributed to peer review, one would expect that it is a relatively optimised process, generally well-understood as a theory and a practice, and stable due to its widespread adoption and acceptance as a method. However, the reality could not be further from the truth. Since its origins, there have been vocal critics about almost every aspect and form of peer review, from its implementation and management, to its wider effects on research culture and the dissemination of scholarship (SI 1) (Smith 2006). However, widespread acceptance of these criticisms, and a desire to improve peer review based on them, can lead to a state of cognitive dissonance for researchers, as criticisms can be interpreted as undermining or challenging the foundations of scholarship itself, as well as the legitimacy of research communities. On the other hand, it is also often considered to be the ‘best that we have’, with scholars remaining frustrated but with the view that alternatives to peer review are always less optimal; irrespective of the diversity of different forms of peer review (Jubb 2016). Thus, scholarly research is in a state of begrudging acceptance of the present state of peer review, despite decades of criticisms and little evidence that it even fulfils the process it is purported to do (for summaries of these criticisms, see Walker and Rocha da Silva 2015; Tennant et al. 2017; Ross-Hellauer 2017a).

What is currently clear is that there is a divergence between how peer review is generally practiced as a multi-dimensional and diverse suite of processes, and how it is commonly regarded as a singular ideologue (Pontille and Torny 2015; Ross-Hellauer 2017b; Casnici Niccolò et al. 2017), despite substantial evidence for a wide range of inherent biases (Lee et al. 2013; Helmer et al. 2017; Kuehn 2017; Tennant 2017; Tomkins, Zhang and Heavlin 2017; Iezzoni 2018). It is now not uncommon to hear views about how to optimise peer review based on smaller population-level studies (e.g. journal- or discipline-level), with polar opposite processes conflictingly hailed as solutions to the same underlying problems (e.g. reviewer blinding versus identification) (Bastian 2017; Tennant 2017). Such diversity of views, while welcomed, has generally inhibited the development of any sort of standardisation behind the definition or the process (Ross-Hellauer 2017a; Allen et al. 2018), which, in turn, has created a fragmented landscape and makes any sort of comparative assessment into the efficacy of peer review problematic (Squazzoni, Grimaldo and Marušić 2017). This has been exacerbated by a general lack of coherence in the implementation of peer review, as well as a paucity of evidence and data sharing on it, making it difficult to draw rigorous conclusions about peer review systems. This conflation of the ideal with the process can also be extremely damaging, as it creates a divergence where trust is expected to fill the gaps, and therefore can be used to undermine the credibility of scholarly research. Thus, the historical controversies surrounding peer review are still reflected in its present systemic state (Thomas 2018).

While the origins of peer-to-peer evaluation can be traced back to the very origins of scholarship (Kronick 1990; Spier 2002; Csiszar 2016; Melinda 2017; Moxham and Fyfe 2017), the advent of what many regard now as the editor-led ‘traditional’ process of ‘peer review’ is only relatively recent, having been established in a piecemeal fashion during the middle of the 20^th^ Century (Zuckerman and Merton 1971; Baldwin 2015). The formalised practice itself only began a century or so before, as part of a community-governed process associated with learned societies and their early scholarly journals (Moxham and Fyfe 2017). Here, contrary to how many academics often view the modern process (Nicholas et al. 2015), peer review was employed mostly to help constructively improve manuscripts by eliminating obvious flaws and gaps in reasoning and improving the rhetorical style and argumentation of articles, rather for any sort of implicit or explicit gatekeeping function. A key here was that peer reviewed scholarly journals became a way of providing scientific legitimacy to learned societies, which was reciprocated by those societies through providing authoritative credibility to those journals. Institutionalisation of the review process took place during the 20^th^ Century, in order to help handle problems with the number of research articles being submitted (i.e. as a gatekeeping or filtering process), as well as to meet increasing demands for expert authority in a research world that was becoming rapidly specialised. One consequence of this was the synonymisation of peer review with scholarly value, which catalysed commercial interest in the process, as it became a way of strengthening journal brands for marketing purposes. An additional effect of this was the effective out-sourcing of the governance of peer review, and the legitimisation that came with it, to commercial entities that operated outside of research communities. Recent developments in ‘Open Peer Review’ (OPR) can largely be viewed as a set of practices to streamline and improve the process in a variety of ways, to help realign the modern practices with the original ideals of progressive collaboration and improving the argumentation style (Melinda 2017; Ross-Hellauer, Deppe and Schmidt 2017; Ross-Hellauer 2017a; Ross-Hellauer 2017b), and to return peer review to its collegial, constructive origins.

Despite the critical importance of peer review in scholarly communication, and considerable recent effort to understand and improve the process, there remain numerous key issues. Some of the main ones include:

- A lack of adequate training and support for researchers in best practices for how to perform peer review (or respond to peer reviews) (Schroter et al. 2004);
- The length of time taken for the peer review process (Bornmann and Daniel 2010; Lyman 2013);
- That valuable contextual information is often lost as review reports remain unpublished (Walker and Rocha da Silva 2015; Ross-Hellauer 2017a);
- What the best operational processes should be for different research communities (Bruce et al. 2016);
- A general lack of rigorous evidence into the functionality of different elements of peer review, including quality (Lee and Moher 2017; Squazzoni, Brezis and Marušić 2017; Squazzoni, Grimaldo and Marušić 2017);
- The relationship between peer review quality and journal quality (Pierson 2018);
- Core competences and standards for editors engaged in peer review (Moher et al. 2017).
- Any form of strategy or consensus on how to address some of the major criticisms levied at peer review (Walker and Rocha da Silva 2015; Tennant et al. 2017; Thomas 2018).

In spite of these challenges, considerable progress in understanding of peer review has been made in recent years and helping to fill in our knowledge gaps about the process. Alongside this, wealth of new platforms and services have emerged that are attempting to resolve some of the socio-technological issues associated with peer review, which has been termed the ‘peer review revolution’ (J. P. Tennant et al. 2017). There has also been an emergence of new interest and data gathering, helping to ignite a new wave of cross-stakeholder discussions and research in to the theory and practice of peer review (Ware 2011; Kovanis et al. 2017; Squazzoni, Brezis and Marušić 2017; Sizo, Lino and Rocha 2018). These ongoing developments are critical to the future of scholarly research, its communication, and the foundational structures of scholarly communities around the world. The purpose of this article is to summarise some of the key elements of the present state of peer review, and hopefully catalyse wider critical discussions and more diverse innovations for its future.

## THE PRESENT STATE OF SCHOLARLY PEER REVIEW

It is estimated that more than 2.5 million English language scientific research publications are now published each year and at a rapidly increasing rate (Ware and Mabe 2015). This creates an incredible burden on the global research workforce, considering that a typical research paper requires 2–3 referees and a handling editor, most of whom act on a volunteer basis for scholarly journals. This has created a state commonly referred to as ‘reviewer fatigue’ (Breuning et al. 2015; C. W. Fox, Albert and Vines 2017), and available evidence suggests that the majority of reviews are performed by a minority of researchers within an increasingly over-burdened system (Lyman 2013; Jubb 2016; Gropp et al. 2017; Kovanis et al. 2017; J. Fox and Petchey 2010; Vines, Rieseberg and Smith 2010). This burden also appears to be unevenly distributed geographically, with Chinese authors reviewing proportionally less articles than western authors (Jubb 2016). Several solutions have been proposed to resolve this state, including how to incentivise more researchers to engage with the review process, with a focus here primarily on quantity rather than quality. This has been directly tied with developments in how to appropriately accredit effort from peer reviewers, such as how to include this work in hiring, promotion, and tenure processes, in which peer review is typically almost entirely absent.

These dual issues of incentivisation and reputation or certification are coupled with a third major issue, effective moderation, which is typically an opaque editorial-controlled function with little standardisation across journals (Moher et al. 2017), but seen as being crucial for injecting any sort of verification or validity into the review process. This aspect is critical for peer review, as typically moderated peer review is seen as the process that differentiates it from other forms of grey literature.

These three factors (incentivisation for engagement, certification and reputation and moderation as a quality control process) are unified by the more complex issue of transparency in peer review, which itself is part of wider changes in the scholarly communication system around the advent of ‘open science’. While there is no single, accepted, unified definition or vision of ‘open science’, one of the core aspects of it revolves around greater transparency throughout the entire research process, including peer review. There are numerous reasons often given for this, such as to combat the ‘reproducibility crisis’, to expose or prevent research misconduct, to introduce greater accountability for researchers, or to increase the verifiability of the research record in order to engender greater public trust for the scientific enterprise (Morey et al. 2016). However, at the present there remains little consensus on the optimal way in which to resolve any of these issues, despite an increasing interest and dedicated research into them.

Much of the current research into peer review focuses on the functionality of the traditional process, its performance and the dimensions of bias (Lee and Moher 2017; Squazzoni, Brezis and Marušić 2017; Squazzoni, Grimaldo and Marušić 2017). As such, improvements to peer review are often centred around these, and are very journal-centric or article-centric by nature—primarily because these are the principle data source. One result of this is that many of the supposed innovations are fairly limited in scope, within the diverse realm of scholarly communication, and tend to be focused within this framework. Consequently, they do little to address the wider issues related to scholarly journals (e.g. journal ranking in research assessment) and articles (e.g. appropriate accreditation) as principle forms of scholarly communication (Brembs, Button, and Munafò 2013).

Despite this generally narrow field of view, there have been numerous recent suggestions about entirely novel methods for scholarly communication and peer review, which have the potential to help solve many of these issues (Priem and Hemminger 2012; Wellen 2013; Nwagwu and Onyancha 2015; Tennant et al. 2017; Schmidt and Gorogh 2017; Heller and Bartling 2014). However, one of the consequences of the way the current peer review system operates is that of cultural inertia (Jónasson 2016), or at least slow rates of adoption, which largely remain in spite of any changes to the surrounding environment. To demonstrate that any new service or platform operates more effectively than current processes, those services must be able to empirically show this in order to obtain any sort of sustainable user base (note, here we mean sustainability for the long-term operation of the platform). However, those same services cannot in turn acquire appropriate usage data for this, as they struggle to acquire the users they need to effectively demonstrate an optimised alternative process needed to incentivise engagement. One key issue here is that the value of peer review as academic capital is often concealed or very difficult to measure, which makes development of incentives to adopt innovative models and practices problematic. The consequence of this is that it all creates a cycle of inertia, where innovations and adoption of those innovations remains fairly stagnant relative to the sustained use of more familiar journal-coupled processes, and progress towards any optimised system remains slow. Such a psychological phenomenon is known as the ‘penguin effect’, whereby a physiological crowd mentality suppresses any experimentation beyond that crowd due to the perception of increased risk and lack of incentive to change (Choi 1994). For now, in 2018, we remain with a scholarly communication system based on a 19^th^ Century process of peer review embedded into a 17^th^ Century method of communication.

## INNOVATIONS IN ‘OPEN PEER REVIEW’

Due to the intrinsic coupling between peer review and scholarly journals, disruptions in peer review are part of a much wider paradigm shift in scholarly communication. Both traditional and newer service vendors are experimenting with a wider range of new models, regarded as a ‘revolutionary phase’ in peer review (Tennant et al. 2017). This has come from a combination of actors, including learned societies and a range of for-profit and non-profit entities, which raises questions around governance structures within scholarly communication and peer review due to the inherent legitimacy associated with the process. One such example is that around responsibility and accountability in peer review, created by the different relationships that exist between researchers and learned societies and scholarly publishers; a factor complicated as some societies now outsource publishing of their journals to commercial entities. As the legitimacy of those institutes is tied to the credibility of the work that they publish, the impact of evolving journal-coupled peer review systems can have quite different implications for their relative standing among research communities. While developments such as Open Access have clearly catalysed innovations in peer review, it is the whole scholarly ecosystem that is evolving in a range of different ways. This has important ramifications for the long-term sustainability of scholarly peer review, and the social aspects that currently govern the different practices.

Perhaps the biggest innovation is that of the increasing trend of ‘open peer review’(Parks and Gunashekar 2017), which itself has become a quite convoluted term (Ross-Hellauer, Deppe and Schmidt 2017; Ross-Hellauer 2017a) within part of broader developments in ‘open science’. It has been diagnosed to refer to seven key aspects of peer review: open identities, open reports, open participation, open interaction, open pre-review manuscripts, open final-version commenting and open platforms (or ‘decoupled review’) (Ross-Hellauer 2017a). Journals and scholarly publishers are now experimenting with various combinations of these traits, in order to find what works best in terms of providing verification, reputation/certification and incentivisation, while balancing transparency within a peer review culture in which opacity is often regarded as the norm, to various degrees (Rooyen et al. 1999; van Rooyen et al. 2010; Parks and Gunashekar 2017; Ross-Hellauer, Deppe and Schmidt 2017; Allen et al. 2018).

In spite of a general ecosystem shift towards openness, it is perhaps fair to say that those who have been most progressive in this regard are the newer ‘born open’ publishers, who have the distinct advantage of firstly being able to build new communities from scratch with different standards, but also not disrupting their own traditions and business models. For example, BioMed Central, Elife, Frontiers, Copernicus, the Self-Journal of Science, PeerJ and F1000 Research represent a range of these ‘born open’ publishers (both for-profit and non-profit) who have adopted different and innovative aspects of open peer review since their beginnings. Very few publishers or platforms seem to fulfill the complete combination of all 7 traits, with exceptions such as ScienceOpen.

Perhaps one of the most critical innovations accompanying this diversification was that of ‘soundness-only’ peer review, often considered a defining trait for megajournals, in which only the scientific rigour of research, not purported novelty or impact, was a deciding factor in publication (Spezi et al. 2017). This principle is more closely aligned with the original learned-society managed process of peer review. Nonetheless, virtually all of these innovations are still centralised around the concept of journals and articles. Even ‘publishing platforms’ are essentially still journals, functionally equivalent to a megajournal (Ross-Hellauer, Schmidt and Kramer 2018), and therefore are only a small step towards migrating into a fully Web-literate and networked mode of peer review and publishing.

### Preprints and post-publication peer review

One of the first platforms launched on the Web was arΧiv in 1991. In numerous sub-disciplines of the physical sciences, mathematics and computer sciences, researchers share non-peer reviewed manuscripts to arΧiv, which currently publishes around 100 000 manuscripts each year (known as preprints or e-prints) (Ginsparg 2016; Pulverer 2016). Here, the purpose is for community-driven cost-effective and rapid communication of research results for collaboration and feedback, which has had differential uptake across the various research disciplines that use arΧiv (Marra 2017). Preprints are currently experiencing an explosive wave of growth in a variety of disciplines, catalysed by a wide range of different tools, platforms and community-level organisations (e.g. ASAPbio, PREreview), often targeted at specific communities that are already adopting preprint services (Tennant et al. 2018). Overlay journals are services that exist by leveraging the existing structures of platforms like arΧiv, with community organised peer review acting as a layer on top of this and the ‘journal’ itself being a collection of links to peer reviewed preprints.

With the ongoing disciplinary expansion in preprint servers (e.g. biorΧiv, multiple servers powered by the Open Science Framework), there is an increasing scope for a number of new overlay journals to be developed, tailored for different research communities. Services such as F1000 Research are similar to preprint platforms, where papers are made available prior to successive iterations of peer review, with manuscripts updated through a simple system of version control. Other services such as PubPeer, PaperHive and ScienceOpen provide a range of post-publication services, typically both on preprints and final version manuscripts.

There remain enormous challenges here in interoperability between vendors, formal recognition of the preprint and ‘post-publication peer review’ process, recognition of the reviews themselves, which can often remain difficult to discover, and then using such reviews to alter published articles, which are often considered to be final (and therefore immutable); a problem exacerbated by the ubiquitous usage of the PDF format and lack of version control. Aggregating reviews from across platforms, and then formalising their recognition as a method of scholarly evaluation is the clear next step here in creating a more continuous peer review and publication workflow (Florian 2012; Kriegeskorte 2012). An interesting consequence of these platforms and services is that initial communication is decoupled from formal journal-based publishing, and new vendors are now increasingly finding ways of integrating peer review into preprint platforms. This has incredibly important consequences on the wider scholarly publishing industry, who must now find ways of justifying their added value, such as journal branding and archiving, once the critical processes of dissemination and peer review have been decoupled from them. Similarly, there is now an increasing responsibility for the research communities adopting preprint platforms to find ways of developing a common infrastructure around preprints, coupled with an explicit scholarly governance model in which accountability is a core trait. Without this, preprints and novel forms of peer review around them will never acquire the same level of legitimacy as journal-based processes.

### Credit for peer review

How to provide and receive appropriate credit for peer review is an ongoing debate. Recently, Crossref, the primary Digital Object Identifier (DOI) provider for scholarly research, announced that review reports could be now registered as part of their services (Lin 2017). This helps to solve the issues of permanent identification and citation of review reports, enabling their wider re-use. Other platforms, such as Publons, provide researchers a way to keep a track of their review record, and integrate this into academic profiles such as ORCID. The focus here is on facilitating credit for peer review, but not actually providing any sort of accreditation themselves—this decision is still based on those in charge of research assessment. While Publons provides a method of allowing authors and other parties to rate review contributions, the primary focus is still on the simple recognition that a review was performed, rather than the intrinsic quality and value of that review. ScienceOpen is a discovery engine that allows researchers to review both preprints and published articles, with each review receiving a CC BY license and Crossref DOI to encourage citation and re-use, and the potential to integrate with Publons and ORCID. There is, therefore, currently a great potential scope of providing more detailed information about peer review quality, in a manner that is further tied to researcher reputation and certification. The main barrier that remains here is the fact that peer review is still largely a closed and secretive process, which inhibits the distribution of any form of credit.

## THE FUTURE OF PEER REVIEW

What would scholarly publishing look like if we rebuilt it from scratch using the tools and knowledge available to us in 2018? This question is not theoretically or conceptually difficult to explore. However, it is problematic often to even discuss, due to the instantaneous resistance that comes because we are talking about disruption of an incredibly complex system adopted by a powerful and thriving industry, and one in which cultural and social norms are deeply embedded across multi-stakeholder processes and institutes. Due to the powerful status of peer review in granting a means of academic capital and prestige, it has gradually evolved to become part of an increasingly bureaucratic and neo-liberal institutional process, which can stifle innovation. Nonetheless, it is a powerful thought process to explore, as essentially it represents a collective vision that most stakeholders in scholarly communications have to streamline the processes, but with extremely different ideas about the time frame that such a vision would be possible to realise in, as well as how to achieve it. Coupled with this, serious consideration is required into whether or not peer review requires a standard, grounded in transparency, in order to be verifiable across a diverse range of communities. This would introduce substantially more rigour into the process, which we should expect from such a critical part of scholarly research.

One key element of this future is the continued decoupling of peer review from journals, through ongoing developments in preprints and community-organised peer review, as discussed above. There is a potential here that researchers begin to see journals as redundant, beyond services such as branding and archiving, and therefore we start to see publishers diversify and unbundle their publishing services. Such could be achieved through the offering or ‘unbundling’ of ‘freemium’ services, such as English-language proofing, copy-editing, type-setting, plagiarism checks and press and media services. Now, large scholarly publishers such as Elsevier are even rebranding as data and analytics companies, perhaps catalysed by the recognition that journals will have significantly less value in the future. However, it is extremely unlikely that the wider scholarly publishing industry will require, or encourage, such a radical shift into services like this, while journal brands are still a dominant factor governing research assessment processes (Brembs, Button, and Munafò 2013). This is perhaps best emphasised by the relatively slow growth of platforms that offer such ‘decoupled’ services, including Peerage of Science and Rubriq, as well as the shutting down of Axios Review in early 2017 (Rajagopalan 2017), in comparison to an otherwise rapidly growing publishing industry. Therefore, the emergence of new services must pay heed to, and where appropriate even influence, wider changes happening in research impact, reputation and evaluation, which strongly influence author choice on publishing venue. This is where the key aspect of certification comes in—it is vastly inappropriate for any new service to discuss researcher incentives for engaging with new models, while not having those incentives formally recognised and valued by those in charge of evaluation and career progression. In order for any aspect of this to achieve progress, there must be a thorough critical discourse about the function of peer review, including knowledge gaps, in order to help the different stakeholders to formulate strong evidence-based policies.

In almost every aspect of the Web, different communities are embracing the power of networks to evaluate diverse forms of information. Scholarly communication is clearly lagging behind this, and in the future, we anticipate the more widespread adoption of collaborative technologies that take advantage of such social processes. These Web-based technologies have the great potential of bridging the presently fragmented landscape of parties interested in peer review (Grimaldo, Marušić and Squazzoni 2018), helping to resolve the general lack of data sharing (Lee and Moher 2017), and providing an accelerated cultural shift towards novel and optimised forms of peer review and research evaluation.

Within different communities and disciplines, there is still a great need for solving issues to do with the exclusivity (Flier 2016), the anonymity, the time and expense (Copiello 2018), the accountability, the subjectivity and bias (Lee et al. 2013), resolving conflicts of interest (Resnik and Elmore 2018), the recognition (Pontille and Torny 2015; Papelis and Petty 2018) and the slow publisher-driven nature of the peer review process (Epstein et al. 2017). Finding the balance between dissemination and validation, reconciled between the different stakeholder groups, will be a key element of this. However, this incredible dimensionality of difficulties should indicate to us that the problems with modern peer review are systemic and encourage us to think outside of the black box of the journal-coupled process to what any modern suite of functions should look like.

As an example of this, Tables 1–3 emphasis the potential different solutions that a hypothetical fully collaborative, Web-enabled process of peer-to-peer review would bring to the many of the issues currently levied at peer review (Priem and Hemminger 2012; Kovanis et al. 2017; Tennant et al. 2017). These are provided in the critical contexts of quality control and moderation (Table 1), certification and reputation (Table 2) and incentives for engagement (Table 3). Only by harmonising all three of these will any successful and sustainable model of peer review be enabled. By illustrating the distinction in this way, it is eminently feasible for any existing or new platform to adopt just one or several of the proposals, rather than a full-scale transformation of the present system. What this represents is a conceptual vision of what is possible, based on existing services, and therefore it is eminently possible for individual factors to be taken up by the present journal-based system. However, as they are all based on traits from existing services (e.g. from GitHub, Wikipedia, or Stack Exchange), it would also be quite possible for them to by all modelled as a single, hybrid construct, if desired.

**Table 1.** Potential future for quality control and moderation.

Traditional	Future
Gatekeeping function as a selective content filter	No gatekeeping, collaboration and constructive criticism define filters
Quality control difficult to measure, with little real evidence of success	Quality control achieved based on consensus, with evaluation based on engagement
Secretive and selective review within a closed system	Self-organised, open and unrestricted communities
Organised around journals and papers	Unrestricted content types and formats
Non-accountable due to ‘black box’ of editorially-controlled process	Elected moderators accountable to their respective communities
Structurally limited and exclusive, usually to 2–3 people	Open participation, with semi-automated review matching
Legitimacy conferred by reputation of brands and editors	Legitimacy provided as a community governed process

**Table 2.** Potential future for certification and reputation.

Traditional	Future
Poorly recognised and rewarded activity for researchers	Performance metrics based on nature and quality of engagement
Difficult to measure due to the opacity of the process	Open, continuous community-based evaluation tied to reputation
Often defaulted to inappropriate higher-level proxies	Granular, revealed at the object and individual levels
Closed process of identification prohibits recognition	Fully transparent by default, tied to academic profiles, and portable
High reviewer turn-down rates, and general frustration for all parties	Expanded reviewer pool with greatly reduced barriers to entry
Level of entry high, based on editorial decision and knowledge	Engagement filters based on reputation within community
Little incentive for those in charge of assessments to care	Appealing for those in charge of assessment due to simplicity

**Table 3.** Potential future incentives for engagement.

Traditional	Future
Shared sense of duty, as a natural altruistic incentive	Same, but with virtual rewards such as points, badges or abilities
Researchers generally feel they receive insufficient credit	Creates an ‘incentive loop’ to encourage maximum engagement
Existing incentives only for engagement, with no focus on quality	‘Reviewing the reviewers’ encourages higher quality engagement
Incentives decoupled from academic reputation or career progression	Coupled to academic records and profiles, and to career advancement
Prestige captured by journals to help define their brands	Establishment of individual prestige as a social process defined by communities

In Table 1, the critical aspect that would define success would be the uptake of any open participation model, such that it was seen as a genuine alternative, not an add on, to formal methods of peer review. These openly collaborative models are already proving highly successful where available, such as with the range of journals published by Copernicus on behalf of the European Geosciences Union (Pöschl and Koop 2008; Pöschl, 2010, 2012). Therefore, there is little stopping any of these individual traits becoming adopted by the present journal-based system, and they could have governance structures maintained by learned societies. This would provide a strong way of shifting towards a fairer and more community-managed processes, as well as embedding additional transparency, accountability, and legitimacy into ‘editorial’ processes. Providing this solution in a sustainable manner across disciplines would require a wider change in culture, based on the recognition that such processes, despite being coupled to journals, have proven to be highly successful in the Geosciences. Other Open Access publishers, such as Frontiers and eLIFE, which also practice forms of collaborative peer review, will be highly important here in demonstrating that open participation can work well in other disciplines. In order to increase the adoption of this, it will be necessary for those publishers to share data on the relative quality of their processes compared with traditional peer review methods in order to demonstrate that it is relatively more effective (or not).

It is impossible to view the potential future model suggested in Table 2 decoupled from the incentives outlined in Table 3, as there is a strong association between researcher reputation and incentives to engage with new processes. This issue is an inherently socio-technical one, and one with which the academic community has been grappling with as part of its culture for some time (Zuckerman and Merton 1971). It is confounded by further problems surrounding values privilege, and bias within scholarly communication and academic cultures. One of the key points here is how to break the association between scholarly journals, arguably a 17^th^ Century mode of communication, and the prestige granted to individuals for publishing in them as a means of academic career progression. So far, this issue has not been concretely resolved, despite decades of understanding the issues associated with it, and numerous alternative proposals. Campaigns, such as the San Francisco Declaration on Research Assessment (DORA), that call for great rigour and transparency in research assessment, do not seem to have had any significant impact on researcher behaviours; if they had, we would have expected to see a weakening of journals as the primary mode of scholarly communication, which has not occurred. Indeed, it is likely that this academic perception of journals as the authoritative source for research, in part due to the apparent verification and certification role that peer review plays when coupled to it, has stifled much of the innovation beyond journal-based peer review in many disciplinary communities (Nicholas et al. 2015). Therefore, one key element to improve this state is that of providing sufficient training and support, particularly for more inexperienced or at-risk reviewers, as well as risk-mitigation strategies, that would enable researchers to be comfortable experimenting with new forms of peer review and scholarly communication.

The key element in Table 3 for incentives is the attempt to capture and define different levels of researcher prestige. At the present, the prestige or reputation of an individual, or individual piece of work, is often tied with journal brands by proxy, but is also an incredibly multi-dimensional concept to comprehend or measure; for example, institutional status, intrinsic biases and privilege and community values and norms. It is difficult to simplify or change this, due to the coupling of prestige with career advancement (Moore et al. 2017); therefore, the key will be demonstrating not that any new method of recognition not only out-performs present models (Kovanis et al. 2017), but that they do so by providing an enriched insight into researcher prestige in a complimentary manner to traditional methods. For example, expanding what it is possible to obtain credit for to include a more diverse suite of research outputs (e.g. data, code and software, images, instructional videos) and coupling this with how that content is digested and engaged with by the wider community should be of considerable interest to those who wish to provide a fairer and more rigorous process of research evaluation, and, in particular, learned societies.

As such, this is why tying additional forms of academic engagement, such as peer review, teaching and public outreach, with certification and reputation (Table 2) will be a critical aspect to consider for any future innovations in this field. This, in turn, relies on getting buy-in from those who are in charge of research assessment, including research funders and hiring committees, which will be pivotal in defining more holistic forms of reputation attainment in order to incentivise more diverse forms of research activity. Indeed, it is likely that a systemic failure to convince institutes as to the value of peer review for academic capital, combined with industrial inertia, has been one of the strongest barriers towards providing sufficient incentives for innovations in peer review. However, with the growth of companies like Publons that seek to provide credit for referees, and their recent acquisition by Clarivate Analytics, we might be encouraged that such reputational incentives might become more firstly increasingly measurable, and secondly more institutionally embedded. In the future, we might expect to see similar initiatives being designed by scholarly communities under their own control, in which they are able to define and regulate certification and accreditation protocols. There is a great potential here to leverage either centralised or decentralised peer-to-peer networks to guide recognition and evaluation in scholarly communication (Hartgerink and van Zelst 2018).

## CONCLUSIONS

The conceptual framework which is outlined here is generally concordant with broader changes in the ‘open science’ movement, reflecting needs for greater transparency in research processes and outputs. While peer review is now an almost exclusively Web-based process now, much of it, and those who adopt it, are still based on non-digital communication norms. The framework outlined here was designed in mind to stimulate further discussion into this issue, and to help increase the reliability of peer review while accounting for some of the caveats associated with innovations in peer review. It also has the potential to help shape a more rigorous method of scholarly evaluation and assessment that could help to simultaneously resolve issues to do with traditional journal-based methods of communication and ranking, something that is critically required for the modern academy (Brembs, Button, and Munafò 2013; Moore et al. 2017; Brembs 2018). The proposal is embedded in principles of open scholarly communication, including inclusivity and open engagement, which are distinct from the traditionally closed and exclusive models of journal-coupled peer review. There is little preventing such changes being adopted as part of a strategic stepwise change within the present publishing industry, to allow for the reformation and adaptation of existing systems, evidence gathering and cultural behaviour to evolve.

All of this potential for innovation in peer review demands that we continue to ask serious questions about the present scholarly communication ecosystem. For example, what are the roles of editors, librarians, and publishers in any proposed or hypothetical future system? What will the impact of any such innovation be on different communities with different social norms, research practices and inherent biases? How do we resolve the tensions between actors who want rapid transformation of peer review, and those who are more conservative or entrenched within the present status quo?

These are not easy questions, and there are certainly not any easy answers. In spite of this, we would like to see continued critical discussion on many of these elements, as well as a removal of the fear to innovate, acknowledgement of any weaknesses, recognition of layers of accountability and the desire to embrace a more diverse thought process around peer review and scholarly communication; all while minimising risk to those who wish to innovate, and making sure that the present power dynamics within scholarly communication are not simply recapitulated in any new system. The key question that unifies the above is why there seems to have been such a low uptake of the different innovative aspects of peer review, when features such as decoupled review, credit enabling and open participation have been around in different forms now for some time. It is likely that there are three primary answers to this, involving a general lack of evidence into the peer review process at different scales, the apparent decoupling of peer review from any sort of formalised recognition for academic career advancement and the above-mentioned perception of risk associated with non-traditional processes of scholarly communication. Therefore, these are the barriers that will likely require most attention in the future of peer review and scholarly communication innovation, and learned societies are perhaps best placed to lead this with the support of their respective communities (Prechelt, Graziotin, and Fernández 2018).

In spite of this, there does however appear to be an emerging wave of momentum and support for disrupting peer review, largely fuelled by social organisations such as ASAPbio, which aims to increase transparency and innovation in the Life Sciences in particular (http://asapbio.org/). This has coincided with a developing understanding of peer review, thanks to the work of initiatives such as PEERE (http://www.peere.org/). The key to maintaining this momentum will be sustained engagement with the different stakeholders to develop a more holistic framework of peer review, in which risk perception is minimised while the advantages are made much more explicit and evidence-based (Rennie 2016).

We anticipate that future discussions and innovations will focus on a number of particular areas:

- The question of sustainability in peer review, what this means for the different actors involved in the process, and how to demonstrate that innovative models are superior to existing ones;
- How to catalyse wider participation in the discussions and innovations in peer review, bearing in mind the incredible social, cultural and practical diversity across disciplines;
- The impact of developments in peer review in different communities, including dimensions of bias and potential socio-technological innovations required to overcome this;
- Whether or not innovations reinforce or disrupt entrenched norms between different research communities;
- A critical appraisal of how to create a more diverse and equitable future for peer review, including the role of peer review in research evaluation processes;
- The role of traditional forms of communication (i.e. journals) and non-community owned publishing platforms, particularly with respect to governance structures;
- How to close the divergence between the original ideal of peer review (and whether this needs to be critically appraised) and the modern practice of it;
- And finally, how Internet-style communication norms can be integrated into peer review, and why our expectations for this to happen seem to be lagging for scholarly publishing and peer review.

While we should not encourage conformation to the status quo in scholarly communication, and a general lack of experimentation, we should also be fully sympathetic towards stakeholders who might not want to see such disruption of scholarly communication norms. Thus, engagement efforts should be focused more on understanding what the reasons for this might be and to use this knowledge to see how to bring what is best for different communities into line with that. There appears to be a general apathetic view towards many aspects of scholarly communication, and it is the responsibility of those who are helping to sculpt this future to maximise participation in it through effective communications. Then, the global scholarly community can collectively help to address the real issues of control and governance of public research. It is our hope that this paper highlights the incredible scope for potential innovations in the future of peer review, and that different communities draw inspiration from that to design optimal systems of research communication.

## Supplementary Material

Supplemental Files
