# Supplementary material for: The state of the art in peer review
Source: FEMS Microbiol Lett. 2018 Aug 23;365(19):fny204. doi: 10.1093/femsle/fny204 (PMC6140953; doi:10.1093/femsle/fny204)
Supplement: Supplemental Files [file fny204_supplemental_files.docx]

| **Authors** | **Title** | **Criticism** |
| --- | --- | --- |
| Margalida et al., 2016 | Improving the peer-review process and editorial quality: key errors escaping the review and editorial process in top scientific journals | Regardless of the mistakes severity, from an editorial point of view, this can be interpreted as (1) a decreasing trend in the quality of peer-review and editorial processes in these journals, or (2) an increase in the trend detection and publication of correction notes. |
| Armstrong, 1997 | Peer review for journals: Evidence on quality control, fairness, and innovation | Efforts by journals to ensure quality and fairness through peer review have not been overly successful. They also reduce the likelihood that important controversial findings will be published, or at least they delay them. |
| Smith, 2006 | Peer review: a flawed process at the heart of science and journals | In addition to being poor at detecting gross defects and almost useless for detecting fraud it is slow, expensive, profligate of academic time, highly subjective, something of a lottery, prone to bias, and easily abused. |
| Jefferson et al., 2002 | Effects of editorial peer review: A systematic review | Given the widespread use of peer review and ist importance, it is surprising that so little is known of ist effects. |
| Peters and Ceci, 1992 | Peer-review practices of psychological journals: The fate of published articles, submitted again | We are sure that there are those who would not wish to see the somewhat delicate machinery of the review system tampered with by a wave of research projects. However, unless we subject the review process, and suggestions for improving it, to experimental analysis to learn more about the variables that do influence peer review, we are left with little to defend it other than faith. |
| Kravitz et al., 2010 | Editorial peer reviewers' recommendations at a general medical journal: Are they reliable and do editors care? | If reviewers cannot regularly agree on whether to recommend rejection or further consideration, the marginal contribution of such summative recommendations may be small, and worse, they may distract from reviewers' primary contribution, which is to improve the reporting – and ultimately the performance – of science. |
| Lloyd, 1990 | Gender factors in reviewer recommendations for manuscript publication | Female reviewers accepted significantly more female-authored (62%) than male-authored (10%) manuscripts. Female-authored manuscripts were accepted significantly more often by female (62%) than by male (21%) reviewers. Information unrelated to the quality of the manuscript appears to have influenced reviewers' decisions. |
| Pöschl, 2012 | Multi-stage open peer review: scientific evaluation integrating the strengths of traditional peer review with the virtues of transparency and self-regulation | The traditional ways of scientific publishing and peer review do not live up to the needs of efficient communication and quality assurance in today’s highly diverse and rapidly developing world of science. |
| Tregenza, 2002 | Gender bias in the refereeing process? | This study suggests that the reviewing process for manuscripts is not ‘institutionally sexist’: there are no overall differences in manuscript acceptance or citation rate relative to gender. However, differences among journals in the acceptance rate of papers relative to gender gives grounds for caution because this pattern is difficult to explain without invoking bias. |
| Fanelli, 2010 | Do pressures to publish increase scientists' bias? An empirical support from US States Data | Since papers reporting positive results attract more interest and are cited more often, journal editors and peer reviewers might tend to favour them, which will further increase the desirability of a positive outcome to researchers, particularly if their careers are evaluated by counting the number of papers listed in their CVs and the impact factor of the journals they are published in. |
| Kornhuber, 1988 | Mehr forschungseffizienz durch objektivere Beurteilung von forschungsleistungen | (the peer review system is)...unreliable, invalid, and harmful to the best type of research - that which is innovative. |
| Daniel, 2004 | Peer review as a target for criticism | Judgmental tendencies on the part of reviewers, as well as publication biases, can constitute a threat to the fairness of the reviewing process. Whether or not this is harmful to the progress of science is a question that has scarcely been investigated. |
| Smith, 2010 | Classical peer review: An empty gun | Yet not only do scientists know little about the evidence on peer review but most continue to believe in peer review, thinking it essential for the progress of science. Ironically, a faith based rather than an evidence based process lies at the heart of science. |
| Ware, 2008 | Peer review in scholarly journals: Perspective of the scholarly community - Results from an international study | There was evidence that peer review is too slow (38% were dissatisfied with peer review times) and that reviewers are overloaded |
| Seeber and Bacchelli, 2017 | Does single blind peer review hinder newcomers? | Overall, the results suggest that by knowing the identity of the authors, reviewers may be biased towards authors that are not sufficiently embedded in their research community. |
| Wang et al., 2016 | Editorial behaviors in peer review | We find out that peer review outcomes are significantly sensitive to different editorial behaviors. With a small fraction (10 %) of biased editors, the quality of accepted papers declines 11 %, which indicates that effects of editorial biased behavior is worse than that of biased reviewers (7 %). |
| Prechelt et al., 2018 | A community's perspective on the status and future of peer review in software engineering | The perception of the current state of software engineering peer review is fairly negative. Also, we found hardly any trend that suggests reviewing will improve by itself over time; the community will have to make explicit efforts. |
| Siler et al., 2015 | Measuring the effectiveness of scientific gatekeeping | Although peer review is widely seen as vital to scientific evaluation, anecdotal evidence abounds of gatekeeping mistakes in leading journals, such as rejecting seminal contributions or accepting mediocre submissions. Systematic evidence regarding the effectiveness—or lack thereof—of scientific gatekeeping is scant, largely because access to rejected manuscripts from journals is rarely available. |
| Siler and Strang, 2016 | Peer review and scholarly originality: Let 1,000 flowers bloom, but don't step on any | Papers that challenged prevailing perspectives were more heavily criticized and more substantially revised than papers that made other sorts of theoretical contributions. |
| Justice et al., 1998 | Does masking author identity improve peer review quality? A randmized control trial | Papers that challenged prevailing perspectives were more heavily criticized and more substantially revised than papers that made other sorts of theoretical contributions. |
| Bruce et al., 2016 | Impact of interventions to improve the quality of peer rreview of biomedical journals: a systematic review and meta-analysis | These results highlight the urgent need to clarify the goal of the peer review process, the definition of a good quality peer review report, and the outcomes that should be used. |
| Fox, 1994 | Scientific misconduct and editorial and peer review processes | Thus the editorial process is fraught with ambiguity in the meaning of review and misconduct, lack of information for detecting or assessing fraud in articles, normative disinclinations to acknowledge and respond to fraud, variations in the type of misconduct by disciplinary area, and unclarity about those to whom an editor is accountable in the case of misconduc |
| Alvesson and Sandberg, 2014 | Habit and Habitus: Boxed-in versus box-breaking research | Researchers safely embedded in the box make sure – as journal editors, reviewers, thesis examiners, assessors of research grant applications, etc. – that the entry costs for moving into a new area are high. Foreign or strange work is treated unfavourably compared to work following the research programmes that make up the boxes. |
| Mahoney, 1977 | Publication prejudices: An experimental study of confirmatory bias in the peer review system | In addition to showing poor interrater agreement, reviewers were strongly biased against manuscripts which reported results contrary to their theoretical perspective. |
